# Scaling Demands of Soccer According to Anthropometric and Physiological Sex Differences: A Fairer Comparison of Men’s and Women’s Soccer

**DOI:** 10.3389/fpsyg.2019.00762

**Published:** 2019-04-09

**Authors:** Arve Vorland Pedersen, Ingvild Merete Aksdal, Ragna Stalsberg

**Affiliations:** ^1^Department of Neuromedicine and Movement Science, Faculty of Medicine and Health Sciences, Norwegian University of Science and Technology, Trondheim, Norway; ^2^Department of Circulation and Medical Imaging, Faculty of Medicine and Health Sciences, Norwegian University of Science and Technology, Trondheim, Norway

**Keywords:** football, gender, stereotypes, biological differences, discrimination

## Abstract

Spectators frequently harass female soccer players, and women’s soccer is frequently compared negatively to men’s soccer by writers who make the comparison without the backing of any data and without taking into account anthropometric and physiological differences between the sexes. This affects female soccer players’ self-confidence negatively and contributes to an undeservedly negative image of women’s soccer. In the present paper, we argue that most differences between men’s and women’s soccer can be explained by women having to adapt to rules and regulations that are suited for men and their physical attributes. Thus, games are much more demanding for women. Furthermore, we argue that if men had to play with a degree of adaptation similar to that which women do today, they would have to alter their style of play radically. As support for our argument, we scale game demands for male and female soccer players according to anthropometric and physiological differences in order to highlight the differences, and use these to predict what would be the most appropriate adaptations. Finally, we show that our predictions are largely supported by the scarce pool of comparable data across the sexes.

## Introduction

*With all respect for what the ladies have done, and they’ve done it fantastically well, you can’t compare men’s and women’s football. Give it up, it’s not even funny. – Zlatan Ibrahimović to the Swedish newspaper* Expressen (see The Guardian, 2013).

The above quote, while being correct enough, came at the end of yet another bout of criticism of female soccer. Even though he stated, “you can’t compare…” the remainder of the quote does exactly that. As Hjelm (2011) correctly pointed out, a statement that men’s and women’s football cannot be compared is self-contradictory, as it presupposes that a comparison has already been made. A more interesting discussion, which can be found in Theberge (1998, 2015) or Sailors (2016), would be whether it is at all relevant to make such comparisons between the sexes.

Even the top female soccer teams will lose to average male teams, and even to boys’ teams (e.g., The Local, 2013). Women’s soccer teams are well aware that they are physically inferior to men’s teams and more similar to boys’ teams, and for that reason, many teams use boys’ teams as sparring partners. This fact is sometimes taken as evidence that the performance level of female soccer is poor. Even worse, soccer fans use such results to harass female soccer players and women’s soccer in general (Hjelseth and Hovden, 2014). Of course, such harassment usually comes from individuals of little expertise and skill (although the source of the above quote is one of the most skilful players in the game).

Even the (later questioned) time of Florence Griffith Joyner in the 100 m sprint at the 1988 Olympic Games (10.49 s) lowering the world record by 0.27 s and setting one that still stands 30 years later (IAAF, 2019), would be smashed by any talented under-20 male sprinter. The best times for these individuals lies at just over 10 s. Furthermore, the best under-18 s can run below 10.2 s (IAAF, 2019). Anyone using such a fact to argue for anything but biological differences between the sexes would be considered quite ignorant. Still, in soccer, many are not able to distinguish between physique and skill, thus giving female soccer an undeservedly bad reputation.

Why is it that so many commentators find it relevant to compare performances directly across the sexes in soccer, while so few would find it worthwhile to compare world records in, say, the high jump (standing currently at 2.45 m for males vs. 2.09 m for females)? Could it be that while the high jump looks similar for males and females – except for the actual height – women and men actually play soccer slightly differently? Alternatively, is it as Hjelm (2011) argued, that while many among the public have personal experience with soccer and thus feel that they could have performed equally as well as the female soccer players, they know very well that they simply *cannot*, themselves, jump 2 m up in the air to negotiate the bar in a high jump?

Although the situation has changed a lot in recent years and women’s soccer has increased its status significantly (Peeters and Elling, 2015; Cardoso de Araújo and Mießen, 2017), the impression still shines through that “real” soccer is played by men and that women’s soccer is inferior. Such impressions will negatively affect girls’ opinions of themselves and their self-confidence as soccer players (Hermann and Vollmeyer, 2016) and may transfer to other domains (Schmader et al., 2008; Chalabaev et al., 2009).

The total number of female soccer players was 30 million in 2014 (FIFA, 2014), and the number is steadily rising. Based on numbers from Europe describing an almost exponential growth (UEFA, 2017), it is not an unreasonable estimate that around 40 million girls and women play soccer worldwide. These players have to live with prejudice and, sometimes, downright misogyny (Hjelseth and Hovden, 2014; Taylor, 2018). The dropout rate from soccer is much higher for girls compared with that for boys (Møllerløkken et al., 2015; Deelen et al., 2018), and one could speculate that one of the reasons why more girls quit soccer is the general negative perception of women’s soccer as inferior.

In fact, it has recently been disclosed that the producers of soccer shoes (boots) do not manufacture their top models in sizes that fit the normal-sized female foot, thus the top players have to use shoes made for children (NRK, 2019). According to a survey conducted by Norwegian Public Broadcasting (NRK, 2019), this fact is perceived by 90% of the players in the premier national women’s league as a sign of prejudice, and 22% of players affirm that they have experienced pain and discomfort due to their shoes not being properly adapted to their feet.

Few attempts have been made to compare men’s and women’s soccer by means of actual data to establish whether there are actually such large differences between the sexes or whether women’s soccer is indeed so poor as often suggested (one notable example being Hjelm, 2011). Others have at least reported data for both sexes on the same match-related variables (Kirkendall, 2007; Gómez et al., 2009; Bradley et al., 2014; Cardoso de Araújo et al., 2018), which is not particularly common. The results have been mixed, partly due to the quality of data, but also due to the studies having been performed under the assumption that the data are actually directly comparable and thus have not been scaled.

The present paper will argue that many – or even most – of the differences between the sexes in the style of play in soccer are due to external physical factors, which lead to logical, strategic adaptations by the female players. Furthermore, it will be argued that male players, given equally challenging demands, would have to adapt somewhat similarly to the way that the females have done.

Firstly, a comparison of relevant anthropometric and physiological variables is presented between the two sexes. Thereafter, these variables are examined in relation to the rules and regulations of the game and scaled according to the anthropometric and physiological sex differences. Finally, the consequences of the differences for the style of play are discussed, followed by speculation about the men’s playing conditions if they were determined by an imagined “anthropometric and physiologically superior sex.”

It is not the scope of the present paper to identify or discuss all the relevant variables but rather to illustrate and exemplify the arguments by presenting a few of the differences. Furthermore, it is not so straightforward to determine which anthropometric or physiological variables would be most relevant to use for a comparison in every case. We have attempted, however, to find fitting variables for each example and to find authoritative sources for establishing the magnitude of sex differences on the included variables.

We would encourage researchers to come up with better variables or to argue for more correct or precise scaling, but we would hold that our argument would stand regardless of the exact magnitude of anthropometric/physiological sex differences. Sometimes, our choices of variables or scaling data may even be too conservative, while it is certainly possible to argue that differences at other times may be exaggerated.

### Some Relevant Anthropometric and Physiological Differences Between the Sexes

In the comparisons seen in Table 1, average values for “ordinary” individuals are used, not sports specific ones involving soccer players. This seems fairer as it reflects pure sex differences and not the current fitness status within the sport, which of course is highly influenced by training. When comparing, for example, endurance values, the relative difference between the sexes is smaller for soccer players, at roughly 12% (Tønnessen et al., 2013; Haugen et al., 2014), compared with a 23% difference for ordinary individuals (Aspenes et al., 2011; Loe et al., 2013). This indicates that female soccer players’ fitness status (more precisely, how much they have improved their fitness relative to the average individual) is better than that of male soccer players. Men’s VO_2max_ values, if similarly enhanced by training, relative to the average ordinary individual would exceed 70 ml⋅kg^-1^⋅min^-1^, which is close to the male average plus one standard deviation (see Table 1).

**Table 1:** Anthropometric and physiological differences across sexes.

	(-2 SD) “inferiors” (+2 SD)	Women (+2 SD)	Men (+2 SD)	“Supermen” (+2 SD)
Height^a^ (also arm span^b^)	(142) 155 (165) cm	168 (180) cm	182 (196) cm	197 (213) cm
Body (forearm-forearm) breadth^c^/5-player wall	40.2 cm/201 cm	46.9 cm/234 cm	54.6 cm/273 cm	63.7 cm/318 cm
Foot length^c^/shoe size	22.15 cm/size 36	24.44 cm/size 39	26.97 cm/size 43	29.76 cm/size 46
Weight at BMI = 23^d^	55 (63) kg	65 (75) kg	76 (95) kg	89 (109) kg
Weight at BMI = 22^e^	54 (64) kg	62 (73) kg	71 (83) kg	81 (96) kg
Speed 100 m (WR)	(12.6) 12.0 (11.4) s	10.9 (10.5) s	9.9 (9.6) s	9.0 (8.7) s
Speed 30 m (soccer)^f^	5.51 (0.8) s	4.84 (0.4) s	4.25 (0.2) s	3.73 (0.1) s
Endurance (VO_2peak_)^g^	34 (48) ml⋅kg^-1^⋅min^-1^	43 (58) ml⋅kg^-1^⋅min^-1^	54 (72) ml⋅kg^-1^⋅min^-1^	68 (90) ml⋅kg^-1^⋅min^-1^
Soccer endurance^h^	50 (52) ml⋅kg^-1^⋅min^-1^	56.5 (59) ml⋅kg^-1^⋅min^-1^	64 (67) ml⋅kg^-1^⋅min^-1^	72 (76) ml⋅kg^-1^⋅min^-1^
% Muscle (average)^i^	28 (41) %	34 (46) %	42 (51) %	52 (57) %
Lower leg strength	(232) 254 (276) N	385 (416) N	584 (627) N	886 (945) N
Jumping height^k^	(13) 23 (33) cm	36 (48) cm	57 (71) cm	90 (104) cm
Kicking velocity (ball speed, instep)^l^	19 (21) m/s	22 (25) m/s	26 (30) m/s	31 (35) m/s


*Actual differences between women and men (+2 SD indicates values for above average players, and -2 SD, similarly, indicates below average players on the team of “inferiors”), and estimated (accordingly scaled) values for individuals equally physically inferior to women as women to men (“inferiors”) and equally superior to men as men to women (“supermen”). ^*a*^Based on data for 20–25-year-olds from the Norwegian Directorate for Health (Helsedirektoratet, 2009). ^*b*^A person’s arm span is roughly the same as the same person’s height (Reeves et al., 1996). ^*c*^Based on data from Gordon et al. (1989). ^*d*^Average BMI for elite European male soccer players, Bloomfield et al. (2005). ^*e*^Argued to be the optimal BMI for female soccer players (Nikolaidis, 2014); also the average BMI for elite female soccer players, (Cardoso de Araújo et al., 2018). ^*f*^Based on data from Baumgart et al. (2018). ^*g*^Aspenes et al. (2011), data for 20–29-year-olds, very similar to Loe et al. (2013) data for 20–29-year-olds. ^*h*^Based on data for national players from Tønnessen et al. (2013) (males) and Haugen et al. (2014) (females). Values extracted from figures. ^*i*^Based on data for 18–29-year-olds in Janssen et al. (2000). ^*j*^Based on data from Kanehisa et al. (1994) data for knee extension, very similar to Miller et al. (1993) data. ^*k*^Scaled accordingly to Patterson and Peterson, 2004; data for 21–25-year-olds. ^*l*^Based on data from Sakamoto et al. (2014).*

For kicking velocity, differences between the sexes are some 18% (Sakamoto et al., 2014). Thus the differences are smaller than for leg strength, which is 33% (Miller et al., 1993), indicating that female soccer players have compensated for some of the strength differences, and furthermore that shooting technique may be more important than mere leg strength. Nevertheless, values for leg strength are used for comparison/scaling because this is consistent with the idea of using values for ordinary individuals – as stated above – and also more comparable to, for example, using VO_2max_ or VO_2peak_ values (the differences between these measures are not important for the present discussion of sex differences, so they will be used interchangeably) for comparing running capacity/endurance.

#### Height/Stature

Males stand, on average, some 10–15 cm taller than females (Garcia and Quintana-Domeque, 2007), with standard deviations of 7 and 6 cm, respectively (Subramanian et al., 2011). The exact difference will vary across countries, but the relative difference is somewhere around 8%. For Norway, an apt example as the home of the authors of the present paper, the difference is 14 cm (see Table 1). Norwegian males are, on average, 1.82 m in stature, while females are 1.68 m, according to the Norwegian Directorate for Health and Social Affairs (Helsedirektoratet, 2009). The standard deviation for males is 7 cm, which makes it probable that quite a few males are closer to 2 m. In fact, assuming a normal distribution of height among soccer players, one would expect 2.5% of them (+2 SD) to be above 1.96 m. The height of the tallest 2.5% of female players would be somewhere around the male average. Bear in mind that Norwegians are among the tallest people in the world (see NCD, 2016). Thus, the problem, although the relative sex difference is similar across countries, will be much greater for countries in which the average height is lower. For example, in the United States, the average height in 2011–2014 was 176.4 cm for males aged 20–29 and 162.9 cm for females of the corresponding ages, albeit with differences of similar relative size across different ethnic groups for both sexes (Fryar et al., 2016). Also, in most countries, even young girls play on the same pitches and use the same goals. In Norway, which is most certainly representative, girls of ages down to 14 years play on same-sized pitches and goalkeepers defend same-sized goals as grown men (NFF, 2019). Thus, they are learning quite different skills and playing a quite different form of soccer from what they will be playing later when their bodies are fully developed, instead of developing skills that are relatively the same as they will be using later on a pitch that is relatively the same (i.e., scaled). We will not be discussing this particular topic in the present article but will instead come back to it in a subsequent paper.

Other anthropometric variables are similarly different between the sexes, although not to the point that the proportions are the same. Simply scaling down a man by 8% would result in a smaller man, but not a woman. The relative difference in size, however, is of somewhat similar magnitude, so scaling by 8% would be close enough for the present argument. For physiological sex differences, on the other hand, which are more variable and generally larger, each variable should definitely be scaled according to actual reported values from scientific papers.

Some relevant sex differences are included in Table 1, including the anthropometric variables: *body width* (forearm-to-forearm), *weight* (adjusted for BMI), and *foot size*. Also treated are the physiological variables: *endurance*, *muscle average*, and *body fat*. Finally, there are the performance differences: *speed*, *jumping height*, and *kicking speed.* The latter differences are, in addition to technique, highly dependent on physiological variables such as lower-leg strength (Patterson and Peterson, 2004; Andersen et al., 2016).

### Rules and Regulations Relative to Anthropometric and Physiological Sex Differences

Soccer, unlike many other sports, possesses rules that are the same for women and men. In fact, handball, ice hockey, volleyball, and basketball all have rules and regulations that are different for the two sexes, such as using balls of smaller sizes/lower weights or various other adaptations of the rules (Theberge, 1998; Andersen et al., 2012). Thus, in soccer, female players have to cope with relatively much tougher demands than males. In the following section, we will present some examples by scaling those variables (rules and regulations) according to the already mentioned sex differences, in order to give the reader an idea of how much they might affect the play.

#### Goal Size

The distance between the posts of a goal is 7.32 m and the distance from the ground to the lower edge of the crossbar is 2.44 m (IFAB, 2018. A reasonable comparison of goal size across the sexes would have to be made relative to the goalkeepers’ height. The average-sized man stands somewhere around 75% of goal height, while an above average-sized man (which is quite normal for goalkeepers) of, say, +1 SD is 78% of goal height (Bloomfield et al., 2005; Ziv and Lidor, 2011). The average heights of goalkeepers in the most recent World Cups for men and women, were 188.9 (5.0) cm, and 173.5 (5.2) cm, respectively. This gives a difference of 8%, which is exactly the same as the general difference between the sexes, but also indicates that female goalkeepers, like their male counterparts, stand one to two standard deviations higher than the average for their sex. Typically, the height of the male goalkeepers was easily accessible via fifa.com (FIFA, 2018a), whereas for the females we had to consult Wikipedia (2018), who have provided the information compiled directly from each association’s official website.

The average-sized woman, on the other hand, is a mere 69% of goal height, while even the taller goalkeepers (+1 SD) would size up to only 72% of goal size. An average female goalkeeper’s reach would be relatively equally shorter as the arm span of each person is proportionate to his or her height (Reeves et al., 1996). Should the goal be scaled down for women according to their relative height, it would be 6.76 m wide and 2.25 m high, while a goal that was scaled up for men to the relative size of the current goal for women would be 7.93 m wide and 2.64 m high.

#### Ball Size and Weight

The standard-sized soccer ball has a circumference of 68–70 cm, with a corresponding weight of 410–450 g (IFAB, 2018). The most relevant variable for comparison of men and women would seem to be foot size or body size, which should be relatively similar in terms of relative difference. Women’s feet are on average some 10.5% shorter than men’s (Gordon et al., 1989), which is similar to the 8% difference in height. This would mean that a fair-sized ball for women would be somewhere between 62 and 64 cm. In fact, this is exactly the same as a size 4 soccer ball which was used for younger age groups and futsal, and previously also in women’s soccer until the early 1990s. Here is a statement made by the United States team after the initial women’s FIFA World Cup in 1991 (FIFA, 2018b), when a size five ball was first used. These thoughts reflect the players’ ambiguity on the matter during a period of a transition of the rules:

*Ball size [no. 5] is acceptable, but the pressure should be lower than for the men*’*s game*. *Because heading skills are not yet fully developed, but also because of differences in physique, women seem to suffer more from headaches*. *Another possibility would be to use a lighter ball, but one without unpredictable flight properties* (*this condition might be difficult to meet*).

For a fair ball weight, the most relevant comparison would probably be leg strength. Here, the ratio of women to men is somewhere around 66% (Miller et al., 1993). A fair soccer ball weight for women would, consequently, be 66% of the median weight of a size five ball (430 g), corresponding to 287 g. The regulation weight of the size four ball is 350–390 g (IFAB, 2018), thus the women’s ball would have to be proportionately even lighter. In fact, 287 g corresponds to a somewhat heavy volleyball (normally 260–280 g, FIVB, 2016). As mentioned, other sports like handball (IHF, 2018) and basketball (FIBA, 2018) use balls of smaller size and mass for women in order to compensate for physiological differences (Andersen et al., 2012). However, we should keep in mind that the weight of the ball could not actually be as low as 287 g, since the flight properties would be much different (as correctly stated in the United States team statement above).

Let us also imagine a reasonable size for the ball to be similarly as unfair for males as the regulation-sized ball is for females. Using the same comparisons (foot size and lower-leg strength), the unfair circumference of the ball would be 76 cm, which is comparable to a basketball (74.93–75.88 cm), of size 7 (FIBA, 2018). The unfair weight would equal some 650 g, which is a little heavier than the same basketball that has a regulation weight of 566.99–623.69 g (FIBA, 2018).

#### Pitch Size and Dimensions

Pitch sizes vary, with the regulations stating that the length of a pitch should be between 90 and 120 m. The width, correspondingly, should range between 45 and 90 m. For international matches, the pitch size should range between 100 × 64 m and 110 × 75 m (IFAB, 2018), with recommended dimensions of 105 × 68 m. Regardless of pitch size, it is a fact that pitches are relatively larger for women. How much larger depends on which measure is used for comparison. One could, for instance, choose to compare the pitch size relative to leg length (i.e., how many strides to cross the pitch), or relative to average height (which corresponds with leg length but is easier to measure). One could compare with reference to endurance (i.e., how much energy is needed to negotiate the pitch), speed (how fast one can run across the pitch), or leg strength (how many kicks are needed to transport the ball across the pitch). All of the above could be argued to be relevant, and one could further argue that a weighted combination would give the fairest comparison. However, two measures could be argued to stand out as more important, namely endurance and leg strength. As can be found in Table 1, females have VO_2peak_/VO_2max_ values that are some 77% of males’ values (Aspenes et al., 2011), or slightly less since Loe et al. (2013) found a 26% sex difference for 20–29 year olds. This means, that a fair relative size of the pitch for female soccer players would be 77% of that for male players. Alternatively, female soccer players would need relative VO_2max_ values that were much higher than males’, and which would place them together with athletes from endurance sports such as long distance running or cross-country skiing (Tønnessen et al., 2015). Alternatively, if leg strength was used for comparison, the women’s pitch area would be 66% of that of the men’s pitch (Miller et al., 1993; Janssen et al., 2000), which corresponds well to actual differences in other areas such as jumping height (Patterson and Peterson, 2004; see Table 1).

It is not only the total pitch area that is unfair to female players. The pitch length and width will also act as constraints due to the mentioned differences in leg strength (Miller et al., 1993). As shown in Table 1, kicking speed is quite different between the sexes (Sakamoto et al., 2014). This difference will affect corner kicks as well as crosses into the box. In order to execute a corner kick or a cross in front of goal with similar speed to that of men, a fair distance for women would be some 66% of that for men. Translated to pitch width, this would mean that the female pitch should be 45 m wide, with the corner spot 22.5 m away from the center of goal (instead of 34 m). A similarly unfair crossing distance for male players would be some 45 m, corresponding to a pitch width of 90 m. Alternatively, female players would have to cross the ball from spaces on the pitch that are relatively closer to the goal, thus functionally narrowing the play relatively, in order to compensate, and to use different strategies for corner kicks than to play the ball directly in front of the goal, as this would involve a lofted kick with a curve that is easier for the goalkeeper and defenders to calculate and hence to defend.

#### Distance to the Wall at Free Kicks (9.15 m)

At free kicks close to the goal, the defending team will usually organize a “wall” of players in order to make it more difficult to score from a direct shot. The goalkeeper defends one side of the goal, while the wall is supposed to defend the other side. In women’s soccer, this wall is lower since players are on average shorter and is thus less effective as a defending strategy. The fact that players are required to stand the same distance away from the ball (9.15 m) as the men, makes it easier to hit the goal by shooting over the wall (due to differences in the required curve of the ball for passing over the wall). Simple maths tells us that the relative effect of the wall is reduced by some 8% (due to the smaller angle of 10.4 degrees compared with 11.25 degrees for men), and that women should be allowed to stand 8.45 m away from the kick for the angle to be equal to that of the men. For a wall of male players to be equally (in-) effective as the female one (same angle), it would have to be placed 10 m away from the kick. The wall is, assuming the same number of players, also narrower as each player is narrower (by some 8.5%, Gordon et al., 1989); hence, a wall of five players will be 20 cm narrower. Consider also that the goal is wider for the female goalkeeper, and the goalkeeper’s reach and jump/dive are shorter. Thus, in order to cover a similarly wide area effectively, one more player would be needed in the wall, leaving one more attacking player free, while the height of the wall cannot be amended at all. In total, the lower and narrower wall makes it much more difficult for female goalkeepers to defend the goal on free kicks.

#### Match Duration

Both men’s and women’s soccer games have a duration of 90 min. Again, a relevant measure for comparison would be the relative endurance between the sexes. As is shown in Table 1, correcting for physiological differences would mean that the women’s game time should be 77% of that for men, thus equalling 70 min. In fact, this was the rule in the earlier days of female soccer when women’s games were actually shorter. In the first World Championship for Women, in 1991, matches were 80 min, a difference of 11%. After the tournament, teams were asked their opinions about the special rules for women. Both finalists, Norway and the United States, when asked about the game time, argued that games should be 90 min, like the men’s, but the United States suggested that there should be unlimited substitution (FIFA, 2018b). At the next WC, in Sweden in 1995, the game time was 90 min. However, teams were allowed up to two timeouts each. For comparison, if the game duration for men was extended so it would be equally as challenging as the women’s game duration is today, it would be 113 min.

When games sometimes go into extra time, another 2 × 15 min are added, and these are played without a break between them. The relative extra time should thus be 24 min (2 × 12 min) to be “fair” for women, and a similarly “unfair” extra time for men would be 37 min (∼2 × 19 min). The total “unfair” game time for men, including extra time, would be 2 × 56.5 min, followed by 2 × 19 min, or in all 151 min.

## Some Consequences of Anthropometric and Physiological Sex Differences for the Style of Play

### Goalkeeping

The female goalkeeper is one player that clearly will suffer from anthropometric/physiological (sex-) differences. It should thus not come as a surprise that this is the position in which female players struggle the most and make the highest number of “mistakes”. Nor would it be surprising that the female goalkeeper is at the receiving end of the majority of harassment. Because female goalkeepers are shorter than their male counterparts are, they will more often let in shots over their heads and not reach shots to either side. Penalties are taken from a distance of 11 m (12 yards), making them hard enough to save for male goalkeepers, especially those shots that are placed close to the goalposts. As female goalkeepers are smaller relative to the goal and have less lower-leg strength (thus, not able to dive equally far or quickly), it is even more difficult for them to save penalties, even though the shots will be relatively weaker (due to the inferior leg strength). Such examples are frequently cited as evidence that the standard of female goalkeepers is inferior (Kirkendall, 2007; see also Hjelseth and Hovden, 2014). However, many such goals are not the result of poor goalkeeping but simply due to female goalkeepers having to defend a relatively much larger goal. The average male goalkeeper, for example, would be able to stop a ball shot with some force to a position just under the crossbar without having to leave the ground, while his average female counterpart would have to jump in order to get enough of her hand behind the ball to deflect it. Furthermore, a female goalkeeper would have to stay much closer to her goal line in order to be able to reach high (lofted) shots with her fingertips so she could push the ball over the crossbar. In all, a female goalkeeper’s relative size leaves much larger spaces undefended in the goal, and taken together with differences in muscle strength she will not be able to reach those spaces fast enough by diving, or jumping, in a manner similar to that of a male goalkeeper.

Interestingly enough, women’s average height today equals that of men’s just before the turn of the 20th century when modern soccer rules were decided, including the goal size. The average height of United Kingdom males born in 1861–1865, who would be adults in 1886 when the basic rules were decided (IFAB, 2018), was 166.25 cm (Hatton and Bray, 2010). Thus, it is not so surprising to find in an old Norwegian book with the title *Football* (Andersen, 1924) a discussion of the height of the goalkeeper when at the time the average height of males in Norway (and in the United Kingdom), was around 170 cm (Hatton and Bray, 2010). Andersen recommended a goalkeeper height of 178–184 cm, which equals the average height at the time plus one-to-two standard deviations. Furthermore, Andersen warned against the goalkeeper leaving his goal because he would then be susceptible to the risk of letting in goals from long-range shots.

In male soccer, the goalkeeper more and more often adopts a sweeper role, in which he can also intercept passes making their way through or over the defense, either with his feet or with his head. The rewards of such a tactic come at the expense of risking making “silly looking” errors. In order to be effective as a sweeper, the goalkeeper must stay as far out from his goal line as possible without leaving the goal open to long-range shots. Thus, there is a clear trade-off between not staying close enough to the goal to stop shots from distance and staying far enough away to intercept through balls. For female goalkeepers, such a trade-off is particularly difficult to cope with as the risk of letting in long-range shots increases proportionally to the goalkeeper’s distance away from the goal. Their relative increased risk arises due to their relative lack of height and because they are relatively slower and need more time to get back to the goal. The cost of errors is similar to that of male goalkeepers who overestimate their ability and try to play sweeper too far out from the goal, but since it will happen more often as the decision is more difficult it has contributed to the female goalkeepers’ reputation for letting in easy goals. Nevertheless, YouTube flourishes with videos of male goalkeepers, including top international ones, making mistakes similar to those of the female goalkeepers.

In men’s soccer, the number of goals per match has decreased in recent years, and it has been increasingly easier for teams to “park the bus” (play extremely defensively) and secure a good result. The steady trend is that fewer goals are scored per game (Njororai, 2013). In the 2018 World Cup in Russia, only 169 goals were scored in the 64 games (2.64 goals per game, FIFA, 2018c). In contrast, in the 1954 World Cup, in Switzerland, an average of 5.38 goals were scored in each game (FIFA, 2018d). Perhaps one should take a closer look at Hertha Berlin manager Dárdai (2018) idea, mentioned in an interview with the newspaper *Märkische Allgemeine*, of increasing the goal size in the men’s game to see more goals scored. Dárdai said that he would rather see games with scores like 4–4 or 5–3 and argued, very similarly to the present argument, that while the average goalkeeper has grown in height over time, the goals have remained the same size, thus they are relatively smaller. Dárdai wanted to increase the goal size by one half meter in height, and a whole meter in width (“Einen halben Meter nach oben und einen halben Meter nach links und rechts”). Doing so would result in goals in the men’s game that are relatively larger than the current ones for women, which relatively equal men’s goals of 7.93 × 2.64 m (see Table 2), but when accounting also for lower-leg strength may perhaps not be so different after all from what female goalkeepers struggle with on a daily basis.

**Table 2:** Rules and regulations with relevance for sex differences in style of play.

	“Fair” for women	Actual situation	“Unfair” for men
Pitch length^a^	84 m (72–96 m)	105 m (90–120 m)	132 m (113–151 m)
Pitch width^a^	54 m (36–72 m)	68 m (45–90 m)	85 m (57–113 m)
Pitch area^a^	5686 m^2^ (∼93 × 61 m)	7140 m^2^ (105 × 68 m)	8967 m^2^ (∼118 × 76m)
Goal width^b^	6.76 m	7.32 m	7.93 m
Goal height^b^	2.25 m	2.44 m	2.64 m
Ball size (diameter)^c^/-weight^d^	62–63 cm/273–300 g	68–70 cm/410–450 g	75–77 cm/621–682 g
Distance for corner kicks^e^	23 m (15–30 m)	34 m (22.5–45 m)	45 m (30–60 m)
Distance to wall at free-kicks^b^	8.45 m	9.15 m	10.7 m
Match duration^a^	72 min	90 min	113 min
Extra time duration^a^	24 min	30 min	38 min
Penalty spot (executer’s point of view)	10 m	11 m	12 m
Penalty spot (goalkeeper’s point of view)^f^	12 m	11 m	10 m


*Actual situation and relative situation when adjusted for anthropometric/physiological differences across sexes. ^*a*^Scaled according to average endurance VO_*2peak/max*_ (±26%), Aspenes et al. (2011); data for 20–29-year-olds, very similar to Loe et al. (2013) data for 20–29-year-olds. ^*b*^Scaled according to average height (±8%), based on data for 20–25-year-olds from the Norwegian Directorate for Health (Helsedirektoratet, 2009). ^*c*^Scaled according to foot-length data from Gordon et al. (1989). ^*d*^Scaled according to differences in lower-leg strength, taken from Miller et al. (1993). ^*e*^Distance from the corner-spot to the center of goal on a pitch of standard international width (68 m); Scaled according to difference in lower leg strength (±33%, Miller et al., 1993). ^*f*^Scaled according to average height (±8%); assuming that lower muscle power plays an equally large role for the executer’s shot and the goalkeeper’s take-off for the dive; however, the disadvantages for the goalkeeper are probably underestimated and should be compensated even more.*

### Passes and Shots

As women have less muscle mass compared to men (Janssen et al., 2000) and are thus not equally as strong (Miller et al., 1993; see Table 1), they use a larger percentage of their total energy to move the ball around, whether on or off the ground. Furthermore, they use relatively more energy moving themselves around. This will consequently lower the tempo of the game, as the alternative would induce fatigue relatively earlier in the game (given that they play the same game length as men) and the quality of play would deteriorate proportionally toward the end of the game (Krustrup et al., 2010).

Another consequence of women’s lesser muscle strength is that the ball will be moved a relatively shorter distance per pass; thus, a team will need more passes, or longer passes, to transport the ball along the length of the pitch. A strategy using more (necessarily shorter) passes would require more time for transportation of the ball across the pitch, which would introduce more possibilities of ball loss, and more shifts in possession. One possible solution would be to move the ball off the ground more often, using so-called lofted passes. This way the ball would travel longer with each pass but would also stay longer in the air due to air resistance (drag), and would be easier to defend, as defenders would have more time available to predict the course of the ball.

Each pass requires a higher percentage of a female player’s maximal force. This will come at the expense of accuracy (as there is a trade-off between force and accuracy; see Fitts, 1954); thus, the probability of errors increases. Alternatively, passes can be made with the same relative force as males with the increased risk of interceptions because the ball velocity will be slower. One could argue that players should then opt for accuracy and sacrifice velocity by applying less force, as (female) opponents are equally slower and not able to intercept the passes anyway. Such a strategy would, however, be hampered by the fact that turf friction remains constant. Unless the turf is wet, the rate at which the ball loses speed would increase almost exponentially. Lofted passes are also more difficult to control for the player receiving the pass and are much more difficult to play on to a teammate or to return using only one touch, which contributes to the slower play.

The same strategy could be argued for shooting using lofted shots more often that are less powerful but more precise (Fitts, 1954). This would seem to be an effective strategy as goalkeepers are smaller and the goal is relatively larger. Being unable to produce force equal to that of males, female players must either come closer to the goal before taking a shot or must choose a different technique such as a lofted shot. Taking into account that goalkeepers are smaller relative to the goal, an effective strategy would be to choose accurate shots over forceful. Placing the ball in the goal, outside the goalkeeper’s reach, is much easier in women’s soccer, both because of the relative goal size (mentioned earlier) and the goalkeepers’ jumping height (Patterson and Peterson, 2004; Ziv and Lidor, 2011); thus, accuracy should be chosen over force.

With less force available, corners and crosses would have to be kicked higher (lofted) instead of being shot rather straight, as is usual in the men’s game. This way the ball stays longer in the air and is easier to defend, due to the goalkeeper’s reach being higher relative to other players’ jumping height. Furthermore, as was argued for long passes, defenders have more time to predict the flight of the ball. However, many of these adaptations would be redundant if the relative ball size was equal to that of men’s soccer. While a smaller ball would definitely be more suited for the female foot, it could be discussed how much lighter the ball should be. There would certainly be a trade-off somewhere as a very light ball, even if hit with great force, would travel slower due to air resistance (drag). Remember also the statement from the United States 1991 WC team, mentioned earlier, where they were clearly aware of the trade-off between ball weight and flight properties. Interestingly enough, they also wanted to use lower pressure, due to the fact that women more often suffer from headaches due to headings. This argument would seem to be supported by the recent findings of Rubin et al. (2018) that women suffer more severe brain injuries from an equal number of headings (see also Pedersen and Stalsberg, 2019). It is not unreasonable to speculate that young female soccer players would avoid headings, and thus develop a different movement pattern because heading the heavier ball is more uncomfortable, which would also affect their heading technique later in their careers.

As it happens, experiments using a smaller, lighter ball in women’s soccer have been performed (Andersen et al., 2012, 2016). According to the developers, speed of play increased, and the ball allowed shots from longer distance as well as other actions described as “impossible with the size five ball” (Eir Soccer, 2019). The basic idea behind designing the ball was that equal foot velocities for men and women would result in equal ball velocities. Thus, it should fly equally far when kicked by a female player as an ordinary soccer ball kicked by a male player with the same relative force (Andersen et al., 2012). True, it was found that female players of different ages are able to kick the lighter ball a longer distance by some 5–6 m compared with the regular ball (Andersen et al., 2016) and by 3–4 m longer for young players (Andersen et al., 2012). Somewhat against expectations, however, no significant differences in style of play were evident. However, players reported a lower degree of perceived exertion in the lower legs. This latter finding, it was argued, may be due to the fact that the ball could be moved around the field with relatively less force which, it was argued, might again reduce injuries.

## What are the Actual Differences in the Style of Play Between Men’s and Women’s Soccer?

While there are relatively few studies comparing men’s and women’s soccer performance directly, we will mention a few results from the relatively scarce data available that are consistent with our hypothesis that differences between the sexes are due to differences in physique.

Female soccer players run less (i.e., shorter total distance) than their male counterparts do, and they play slower. Not only are they unable to sprint as fast as male players (Baumgart et al., 2018), but females also spend longer periods of the game at lower velocities (Bradley et al., 2014). Despite this, female players get more fatigued earlier in the game (Mohr et al., 2005; Krustrup et al., 2010), and their performance decreases more, relative to males, in the second half (Bradley et al., 2014), as well as within the latter quarters of each half.

Female players experience more ball losses than male players do, and they have a lower percentage of successful passes (Bradley et al., 2014). More goals are scored from long-range shots (from outside the penalty area) in female soccer compared with male soccer (Kirkendall, 2007). Furthermore, female players take more shots after team-plays, while male players’ shots are more often preceded by individual play (Gómez et al., 2009). The total number of shots is similar across the sexes, but female players have more shots on goal. The latter, argued Gómez et al. (2009), may be due to the fact that male defenders are able to clear more shots compared with their female counterparts. The conversion rate (goals per shot) of female players is higher, due to many more shots being saved by the male goalkeepers (Gómez et al., 2009). The fact that female players take more shots from long range compared with male players may be argued to be inconsistent with predictions based on differences in lower-leg strength, but it is consistent with predictions based on the goalkeeper’s size relative to the goal.

To give a fair comparison of female and male soccer, one would have to scale values according to the relative physiological capacities and establish, for example, gender specific thresholds for running intensity, as was suggested in Bradley et al. (2014). Thus, it would seem fair to compare the female players’ running distances at above 12 km h^-1^ with the male players’ corresponding distances above 15 km h^-1^. Furthermore, as was noted by Kirkendall (2007), there is an overlap in running distance between the sexes in that while women average 10 km per game, men’s distances range from 10 to 14 km, thus some female players run as much as many male players.

Modern styles of play often involve a high-pressing system. Pressing high requires extreme endurance because of the relatively larger pitch. Such tactics, if applied by women’s teams, would necessitate a high (-er than for males) amount of physical conditioning (which turns out to be the case judging from the relatively higher VO_2max_ values). Such training might come at the expense of technical skills practice (see Kirkendall, 2007).

## A Thought Experiment

Let us now imagine a “third sex,” or the discovery of a different species, with the same relative anthropometric/physiological advantages over men that men have over women. What might this comparison look like? As can be seen in Table 2, the average player of such a team would be just below 2 m in height, with the occasional player (+2 SD) of over 2.10 m, among them an extremely quick goalkeeper of 2.15 m, with an arm span the same as his height. The “supermen,” as we may call them, would weigh about 10 kg more than men, of which most would be muscle. “Supermen” would average VO_2max_ values of 77 ml⋅kg^-1^⋅min^-1^ with some values as high as in the mid-80 s. Such values, as mentioned, are comparable with the top athletes in endurance sports.

While male players, with their average 30 m sprint times of 4.25 s and best times of just over 4 s (Baumgart et al., 2018) can easily outsprint their female counterparts who are, on average, 0.6 s slower, the “supermen” would average their 30 m sprints in some 3.75 s. Their fastest players would run the distance in closer to 3.7 s. Of course, as for the difference between males and females, even the slowest players from the team of “supermen” would beat the average player from the men’s team, and most all would beat the fastest man.

A game between men and “supermen” would of course be played according to rules and regulations suited for the supermen, with a game time of 113 min on a pitch that was some 118 × 76 m (8967 m^2^) or even larger. We should bear in mind that this size is still within the actual regulations (IFAB, 2018), which state that the maximum pitch length is 120 m and the maximum pitch width is 90 m.

We will not have to be equally imaginative to find individuals similarly inferior to average women as average women to average men. As it happens, the “inferiors” in Table 2, with a height of 155 cm, and weight of 54 kg (at BMI = 22), are comparable in body size to 12–13-year-olds, whether boys or girls, as they are more or less equal in size at this age (see Hamill et al., 1979). In other variables more directly relevant to soccer, for example, speed and power, the adult females may be more similar to 14-year-old boys (Baumgart et al., 2018). This latter observation effectively closes our argument, as it explains why women’s teams play against 15–16-year-old boys (who are physically superior but not by much) and why, as mentioned in the beginning of this article, the Swedish women’s national team could lose against a decent team of 17-year-old boys.

## Concluding Remarks

The present authors will not advocate changing the rules and regulations of soccer. That is the job of FIFA (or more specifically, IFAB) to decide and for female players and their coaches and leaders to argue for, should they wish to do so. The intention is rather to point at the obvious (but still not sufficiently recognized) anthropometric/physiological differences, and argue that differences in style of play between men’s and women’s soccer are logical and strategic adaptations to those differences. Thus, one should not expect women’s soccer to be exactly like men’s. Should one, however, wish to see women’s soccer look more like men’s it would require quite a number of regulation changes. The pitch would have to be some 93 × 61 m, with goals of 6.78 × 2.26 m. The ball would have to be a size 4, slightly heavier than a volleyball (note however previous arguments about flight properties), and the duration of the game would have to be 70 min.

What we want people to understand, is that the present situation for women is comparable to men playing on a 118 × 76 m pitch (and bear in mind that this is a conservative estimate), with goals of 7.93 × 2.64 m, and playing with a ball very similar in size and weight to a basketball. The game would last 113 min (ca. 2 × 56 min), which is comparable to that of a game going into extra time, especially when taking into account that there would be no extra break at 90 min. (Remember that should such a prolonged game extend into extra time, another 38 min would await the players.) Even without the imagined “supermen” as opponents, this would be a very interesting game, and one that the authors of the present paper would certainly encourage broadcasters to organize and televise.

An even better solution than making rule changes, however, would be to enjoy the game as it is today, with its small differences between the sexes, and perhaps work to increase the competence level of the spectators.

## Data Availability

All datasets analyzed for this study are included in the manuscript and the Supplementary Files.

## Author Contributions

All authors made substantial contributions to the paper, participated in drafting and revising the article, and approved the final version for publication. AVP conceived the idea of the paper.

## Conflict of Interest Statement

The authors declare that the research was conducted in the absence of any commercial or financial relationships that could be construed as a potential conflict of interest.
